# Extreme thiopurine metabolite accumulation and life-threatening myelotoxicity due to cumulative pharmacogenetic susceptibility in Crohn’s disease

**DOI:** 10.1093/gastro/goag105

**Published:** 2026-10-06

**Authors:** Julien Moragny, Emeline Delabre, Florine Foré, Youssef Bennis, Valérie Gras, Mohamad Houssam Al Balkhi

**Affiliations:** Pharmacovigilance Centre, Department of Clinical Pharmacology, Amiens-Picardie University Hospital, F-80054, Amiens, France; Pharmacovigilance Centre, Department of Clinical Pharmacology, Amiens-Picardie University Hospital, F-80054, Amiens, France; Department of Pediatric Onco-hematology, Amiens-Picardie University Hospital, F-80054, Amiens, France; Pharmacovigilance Centre, Department of Clinical Pharmacology, Amiens-Picardie University Hospital, F-80054, Amiens, France; Pharmacovigilance Centre, Department of Clinical Pharmacology, Amiens-Picardie University Hospital, F-80054, Amiens, France; Pharmacovigilance Centre, Department of Clinical Pharmacology, Amiens-Picardie University Hospital, F-80054, Amiens, France

## Introduction

Thiopurines remain widely used in inflammatory bowel disease (IBD), yet their narrow therapeutic index variability predisposes genetically susceptible individuals to severe hematological toxicity [1, 2]. Excessive intracellular accumulation of active erythrocyte 6-thioguanine nucleotide (6-TGN) mediates thiopurine-induced myelotoxicity [2, 3]. Variants in thiopurine S-methyltransferase (TPMT) and nudix hydrolase 15 (NUDT15) are well-established determinants of thiopurine-induced myelotoxicity [3]. Combination therapy with infliximab and azathioprine is frequently used in Crohn’s disease to enhance treatment durability and reduce immunogenicity [1], but may increase myelosuppression risk in genetically susceptible patients. Reports describing the coexistence of multiple pharmacogenetic defects remain rare. We report life-threatening thiopurine toxicity resulting from cumulative pharmacogenetic susceptibility involving TPMT, NUDT15, and ITPA variants, illustrating how polygenic impairment may produce extreme toxicity exceeding individual variant effects and highlighting the limitations of relying solely on hematological thresholds during early monitoring.

## Case report

A 16-year-old male of European ancestry with newly diagnosed ileocolonic Crohn’s disease and moderate disease activity was started on infliximab. Azathioprine (100 mg/day; ∼1.5 mg/kg) was subsequently introduced to reduce immunogenicity. The first infliximab infusion was administered 9 weeks before azathioprine initiation (Week −9), followed by infusions at Weeks +1 and +5 relative to azathioprine start (Week 0) (Fig. 1). Pretreatment TPMT and NUDT15 genotyping was not performed. Laboratory testing immediately prior to azathioprine initiation (Week 0) showed hemoglobin 12.5 g/dL, white blood cell count (WBC) 4.6 × 10^9^/L, absolute neutrophil count (ANC) 1.3 × 10^9^/L, and platelets 196 × 10^9^/L. A subsequent measurement at Week 1 showed an ANC of 3.18 × 10^9^/L, likely reflecting biological variability and post-inflammatory recovery.

**Figure 1:**
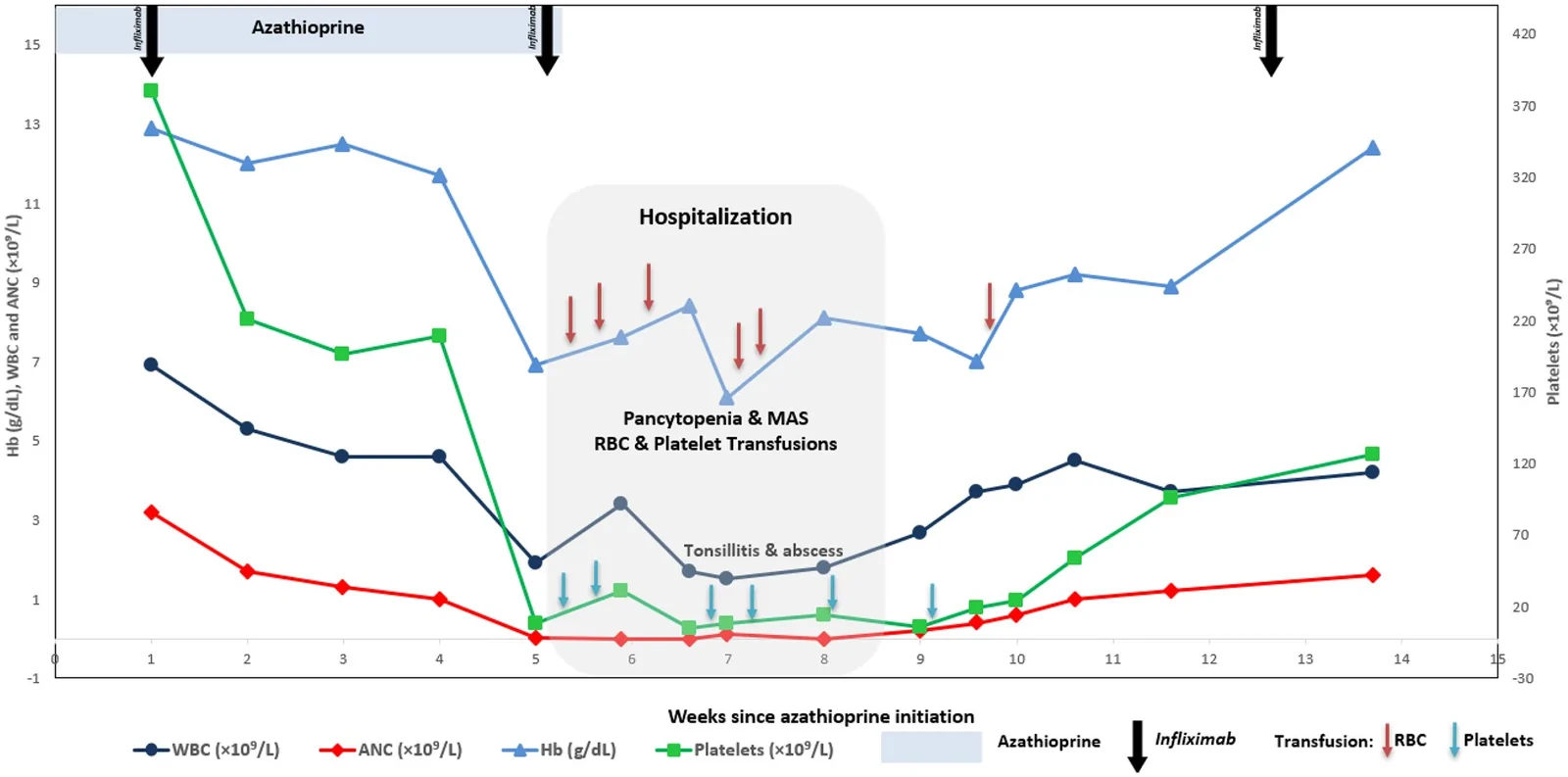
Hematological evolution during and after azathioprine therapy. Temporal evolution of hemoglobin (Hb), white blood cell count (WBC), absolute neutrophil count (ANC), and platelet count after azathioprine initiation (Week 0). A progressive decline in neutrophil counts occurred during Weeks 1–4 and preceded abrupt pancytopenia at Week 5. Profound neutropenia persisted until Week 8, followed by gradual hematological recovery. The shaded area indicates hospitalization with severe pancytopenia requiring red blood cell (RBC) and platelet transfusions. Infliximab infusions are indicated by black arrows and azathioprine exposure by the shaded bar.

During Weeks 1–4 of azathioprine therapy, a progressive decline in neutrophil counts developed: ANC declined from 3.18 × 10^9^/L at Week 1 to 1.0 × 10^9^/L at Week 4. Despite this progressive decline, azathioprine was continued. No hematological reassessment was performed between Weeks 4 and 5 (Fig. 1). At Week 5, abrupt pancytopenia occurred (WBC 1.9 × 10^9^/L; ANC 0.031 × 10^9^/L; hemoglobin 6.9 g/dL; platelets 8 × 10^9^/L). Azathioprine was immediately discontinued. At Week 6, C-reactive protein was 364 mg/L and ferritin 1,187 µg/L. Given the temporal sequence, infliximab was considered unlikely to have contributed to the pancytopenia. Between Weeks 6 and 7, the hematological nadir occurred, with ANC 0 × 10^9^/L and platelets 5 × 10^9^/L. Profound neutropenia (ANC ≤ 0.1 × 10^9^/L) persisted through Week 8, followed by gradual recovery. Granulocyte colony-stimulating factor (G-CSF) was not administered. Supportive management with azathioprine withdrawal, broad-spectrum antibiotics, transfusional support, and close monitoring was favored, as the role of G-CSF in thiopurine-induced marrow suppression remains uncertain.

Bone marrow aspiration demonstrated hypocellularity with hemophagocytosis consistent with drug-induced myelotoxicity with secondary hemophagocytosis rather than primary HLH [4]. Extensive infectious and autoimmune investigations were negative. Erythrocyte 6-TGN concentrations at Week 5 were markedly elevated (>7,710 pmol/8 × 10^8^ red blood cells (RBC); therapeutic range 235–400 pmol/8 × 10^8^ RBC), far exceeding concentrations typically associated with leukopenia in IBD (∼200–400 pmol/8 × 10^8^ RBC) [5], providing direct pharmacological evidence of thiopurine toxicity. Although analytical error was considered, the result was biologically consistent with profound myelotoxicity and the subsequent decline after azathioprine withdrawal (545.4 pmol/8 × 10^8^ RBC at Week 9; 2.96 pmol/8 × 10^8^ RBC by Week 13). The patient required antibiotics, intensive supportive care, and repeated transfusions. Hematological recovery was prolonged and complicated by bacterial tonsillitis with peritonsillar abscess. Following azathioprine discontinuation, erythrocyte 6-TGN concentrations rapidly declined, reaching 545.4 pmol/8 × 10^8^ RBC at Week 9 and 2.96 pmol/8 × 10^8^ RBC by Week 13. Hematological recovery was gradual, with normalization of the ANC by Week 14 (1.6 × 10^9^/L). Infliximab was resumed at Week 13 without recurrence of cytopenias. At 12-month follow-up, the patient remained in clinical remission on infliximab monotherapy. Pharmacogenetic analysis revealed complete TPMT deficiency due to compound heterozygosity for loss-of-function alleles (TPMT*2 and TPMT*3A), heterozygosity for the nonfunctional NUDT15*3 allele, and heterozygosity for the inactivating ITPA IVS2 + 21A > C variant. Azathioprine was permanently contraindicated.

## Discussion and conclusions

This case illustrates life-threatening thiopurine toxicity associated with cumulative pharmacogenetic susceptibility. Complete TPMT deficiency (prevalence 0.03%–0.5%) impairs thiopurine inactivation [3]. Combined TPMT and NUDT15 variants are associated with early and severe myelotoxicity, often within the first weeks of treatment [6]. Markedly elevated 6-TGN concentrations were consistent with the pharmacogenetic profile and profound marrow suppression. The reintroduction of infliximab without cytopenia recurrence further supports azathioprine as the primary causative agent. The progressive neutrophil decline during Weeks 1–4 represented an early warning signal and a missed opportunity for prevention. In the present case, the absence of hematological reassessment between Weeks 4 and 5 delayed recognition and earlier azathioprine interruption might have limited the pancytopenia severity. Even sustained neutrophil declines within the normal range should prompt immediate reassessment, particularly when the pharmacogenetic status is unknown. Although thiopurine doses are generally lower in IBD than in oncology, monitoring is often less intensive, reinforcing the importance of pretreatment pharmacogenetic testing and early laboratory surveillance [2].

This case supports a polygenic model in which combined TPMT, NUDT15, and ITPA variants may lead to extreme intracellular accumulation of cytotoxic thioguanine nucleotides. Although the contribution of ITPA variants in IBD remains debated and is not incorporated into current dosing guidelines [3, 7], several studies have associated the ITPA IVS2 + 21A > C variant with thiopurine-related myelotoxicity [7, 8]. More recently, Salazar *et al.* identified ITPA IVS2 + 21A > C as an independent predictor of thiopurine-related adverse events and myelotoxicity in Crohn’s disease patients with intermediate–normal TPMT activity [9]. However, replication across IBD cohorts remains inconsistent and ethnic variability together with interactions between TPMT and NUDT15 variants may explain the discrepancies [8, 9]. Accordingly, ITPA IVS2 + 21A > C should be interpreted cautiously as a potential modifier of toxicity risk rather than an established determinant of severe toxicity. G-CSF was not administered and recovery occurred progressively with supportive care alone, although its role in thiopurine-induced marrow suppression remains uncertain. To our knowledge, this exact combination of TPMT deficiency, NUDT15*3 heterozygosity, and ITPA IVS2 + 21A > C has not previously been reported in pediatric Crohn’s disease. Current guidelines recommend TPMT and NUDT15 testing before thiopurine initiation [3], although implementation remains variable in routine clinical practice. This case highlights the importance of pharmacogenetic testing and close early hematological monitoring during thiopurine initiation.
